# Zika virus infection estimates, Mexico

**DOI:** 10.2471/BLT.17.201004

**Published:** 2018-02-28

**Authors:** Juan Eugenio Hernández-Ávila, Lina Sofía Palacio-Mejía, Hugo López-Gatell, Celia M Alpuche-Aranda, Diana Molina-Vélez, Leonel González-González, Mauricio Hernández-Ávila

**Affiliations:** a National Institute of Public Health, Cuernavaca, Mexico.; b National Council for Science and Technology, Mexico City, Mexico.; c Centro Universitario de los Altos, Universidad de Guadalajara, Carretera a Yahualica, Km 7.5, Tepatitlán de Morelos, Jalisco 47600, Mexico.

## Abstract

**Objective:**

To assess the magnitude of the Mexican epidemic of Zika virus infection and the associated risk of microcephaly.

**Methods:**

From the reported number of laboratory-confirmed symptomatic infections among pregnant women and the relevant birth rate, we estimated the number of symptomatic cases of infection that occurred in Mexico between 25 November 2015, when the first confirmed Mexican case was reported, and 20 August 2016. We used data from the birth certificates to compare mean monthly incidences of congenital microcephaly before (1 January 2010–30 November 2015) and after (1 December 2015–30 September 2017) the introduction of Zika virus, stratifying the data according to whether the mother’s place of residence was at an altitude of at least 2200 m above sea level. We used Poisson interrupted time series, statistical modelling and graphical analyses.

**Findings:**

Our estimated number of symptomatic cases of infection that may have occurred in the general population of Mexico between 25 November 2015 and 20 August 2016, 60 172, was 7.3-fold higher than the corresponding number of reported cases. The monthly numbers of microcephaly cases per 100 000 live births were significantly higher after the introduction of the virus than before (incidence rate ratio, IRR: 2.9; 95% confidence interval, CI: 2.3 to 3.6), especially among the babies of women living at altitudes below 2200 m (IRR: 3.4; 95% CI: 2.9 to 3.9).

**Conclusion:**

The Mexican epidemic appears to be much larger than indicated by estimates based solely on counts of laboratory-confirmed cases, and to be associated with significantly increased risk of microcephaly.

## Résumé

**Objectif:**

Évaluer l'ampleur de l'épidémie d'infections à virus Zika au Mexique et estimer le risque associé de microcéphalie.

**Méthodes:**

En utilisant le nombre de cas signalés, confirmés en laboratoire, d'infections symptomatiques de femmes enceintes et le taux de natalité correspondant, nous avons fait une estimation du nombre de cas symptomatiques d'infections survenus au Mexique entre le 25 novembre 2015 (date de signalement du premier cas confirmé au Mexique) et le 20 août 2016. Nous avons utilisé les données extraites des certificats de naissance afin de comparer les incidences mensuelles de microcéphalie congénitale, antérieures et postérieures à l'introduction du virus Zika (respectivement du 01 janvier 2010 au 30 novembre 2015 et du 01 décembre 2015 au 30 septembre 2017), en stratifiant les données selon que le lieu de résidence de la mère se situait en dessous ou à partir de 2200 mètres d'altitude au-dessus du niveau de la mer. Nous avons réalisé une analyse en séries temporelles interrompues à l'aide d'un modèle de régression de Poisson, des modélisations statistiques et des analyses graphiques.

**Résultats:**

Notre estimation du nombre de cas symptomatiques d'infections susceptibles d’être survenus dans la population générale du Mexique entre le 25 novembre 2015 et le 20 août 2016 (à savoir 60 172) s'avère 7,3 fois supérieure au nombre de cas effectivement signalés pour la même période. Les chiffres mensuels des cas de microcéphalie pour 100 000 naissances vivantes sont considérablement plus élevés après l'introduction du virus qu'avant l'introduction du virus (rapport des taux d'incidence, RTI: 2,9; intervalle de confiance (IC) de 95%: 2,3–3,6), notamment chez les enfants nés de mères vivant à des altitudes inférieures à 2200 mètres (RTI: 3,4; IC 95%: 2,9–3,9).

**Conclusion:**

L'épidémie mexicaine semble bien plus importante que ce qu'indiquent les estimations uniquement fondées sur les cas confirmés en laboratoire et semble être associée à un risque considérablement plus élevé de microcéphalie.****

## Resumen

**Objetivo:**

Evaluar la magnitud de la epidemia de infección del virus Zika y el riesgo asociado de microcefalia en México.

**Métodos:**

Del número de infecciones sintomáticas reportadas en laboratorio entre mujeres embarazadas y la tasa de nacimiento relevante, estimamos el número de casos de infección sintomática que ocurrieron en México entre el 25 de noviembre de 2015, cuando se confirmó el primer caso mexicano, y el 20 de agosto de 2016. Usamos la información de los certificados de nacimiento para comparar incidencias mensuales significativas de microcefalia antes (1 de enero de 2010-30 de noviembre de 2015) y después (1 de diciembre 2015-30 de septiembre de 2017) de la introducción del virus Zika, estratificando la información de acuerdo a si el lugar de residencia de la madre estaba a una altitud de al menos 2200 m sobre el nivel del mar. Se usaron series temporales interrumpidas de Poisson, modelos estadísticos y análisis de gráficos.

**Resultados:**

Nuestro número estimado de casos de infección sintomática que podría haber ocurrido en la población general en México entre el 25 de noviembre de 2015 y el 20 de agosto de 2016, 60.172, fue 7,3 veces más elevado que el número correspondiente de casos informados. El número mensual de casos de microcefalia por cada 100.000 nacimientos vivos fue significativamente más alto después de la introducción del virus que antes (razón de tasas de incidencia, IRR: 2,9; intervalo de confianza, IC, del 95%: 2.3 a 3.6) especialmente entre bebés de mujeres viviendo en altitudes menores a 2200 m. (IRR: 3,4; IC del 95%: 2.9 a 3.9).

**Conclusión:**

La epidemia mexicana parece ser mucho más grande que las estimaciones indicadas basadas solamente en la contabilización de casos confirmados por laboratorio y está asociada con un incremento significativo de riesgo de microcefalia.

## ملخص

**الغرض:**

تقييم حجم الإصابة بوباء فيروس زيكا في المكسيك والمخاطر المصاحبة للإصابة بالصعل (صِغر الرأس).

**الطريقة:**

من خلال استخدامنا للأعداد الواردة حول الإصابات المصحوبة بالأعراض والمؤكدة مختبريًا بين السيدات الحوامل ومعدل المواليد ذي الصلة، قمنا بتقدير عدد حالات الإصابة المصحوبة بالأعراض التي ظهرت في المكسيك ما بين 25 من نوفمبر/تشرين الثاني لعام 2015 حيث سُجلت أولى حالات الإصابة المؤكدة و20 أغسطس/آب من عام 2016. وقد استخدمنا البيانات الواردة في شهادات الميلاد للمقارنة بين المتوسط الشهري للإصابة بالصعل الخلقي قبل (1 يناير/كانون الثاني 2010 وحتى 30 نوفمبر/تشرين الثاني 2015) وبعد ظهور فيروس زيكا (1 ديسمبر/كانون الأول 2015 حتى 30 سبتمبر/أيلول 2017)، وقمنا بتقسيم البيانات إلى شرائح تحليلية وفقًا لمكان إقامة الأم إذا كان على ارتفاع 2200 متر على الأقل فوق سطح البحر. وقد استخدمنا أسلوب السلاسل الزمنية المتقطِّعة لبواسون، ونماذج إحصائية، وتحليلات بيانية.

**النتائج:**

ارتفع العدد المقدّر من جانبنا والبالغ 60172 لحالات الإصابة ****المصحوبة بالأعراض والتي قد تكون قد أصابت عموم السكان في المكسيك في الفترة ما بين 25 شهر نوفمبر/تشرين الثاني عام 2015 و20 أغسطس/آب عام 2016 بمقدار 7.3 أضعاف عن الأعداد المقابلة للحالات المسجلة. وقد ارتفعت الأعداد الشهرية لحالات الإصابة بالصعل بدرجة ملحوظة لكل 100 ألف من المواليد الأحياء بعد ظهور الفيروس مقارنة بقبل ظهوره (نسبة معدل وقوع الحالة (IRR): 2.9، بنسبة أرجحية مقدارها ‏95%: 2.3 إلى 3.6) وقد ظهر ذلك بشكل خاص بين المواليد للنساء اللاتي يعيشن على ارتفاع أقل من 2200 متر (نسبة معدل وقوع الحالة (IRR): 3.4، بنسبة أرجحية مقدارها ‏95‏‏%: 2.9 إلى 3.9).

**الاستنتاج:**

من الواضح أن الوباء في المكسيك أكثر انتشارًا بكثير مما تشير التقديرات والتي تعتمد فقط على تعداد حالات الإصابة المؤكدة مختبريًا، ويصاحب الوباء كذلك الخطر المتزايد بشكل ملحوظ للإصابة بالصعل.

## 摘要

**目的:**

旨在评估墨西哥寨卡病毒的感染率以及相关的头小畸型风险。

**方法:**

据报告，经实验室确诊出现症状的孕妇感染案例数和相关出生率，我们估计了 2015 年 11 月 25 日（首例报告确诊的墨西哥案例）至 2016 年 8 月 20 日之间在墨西哥发生的出现症状的感染案例量。我们采用了出生证明上的数据以比较寨卡病毒传入前（2010 年 1 月 1 日至 2015 年 11 月 30 日）后（2015 年 12 月 1 日至 2017 年 9 月 30 日）先天性头小畸型平均每月的发病率，并根据母亲的居住地是否至少位于海拔 2200 米的高度上对数据进行分层。我们采用了间断泊松时间序列、统计建模和图像分析。

**结果:**

我们对 2015 年 11 月 25 日至 2016 年 8 月 20 日在墨西哥一般人群间发生的出现症状的感染案例的估计量（60 172 例）较相应的报告案例量高出 7.3 倍。该病毒传入后，每月每 100 000 例活产婴发生头小畸型的数量明显高于传入前（发病率，IRR：2.9；95% 置信区间，CI：2.3 至 3.6），尤其多发于母亲生活在海拔 2200 米以下高度的婴儿间 (IRR: 3.4; 95% CI: 2.9 至 3.9)。

**结论:**

完全根据实验室确诊案例得出的估算值表明，墨西哥的病情传播范围似乎更广，而且与头小畸形风险的显著增加相关。****

## Резюме

**Цель:**

Оценить масштабы эпидемии инфекции, вызываемой вирусом Зика, в Мексике и связанный с ней риск микроцефалии.

**Методы:**

На основе количества зарегистрированных, лабораторно подтвержденных случаев симптоматической инфекции среди беременных женщин и соответствующего коэффициента рождаемости авторы установили число случаев симптоматической инфекции, которые возникли в Мексике в период с 25 ноября 2015 года, когда был зарегистрирован первый подтвержденный случай заболевания в стране, по 20 августа 2016 года. Авторы использовали данные свидетельств о рождении для сравнения среднемесячного количества случаев врожденной микроцефалии до (с 1 января 2010 года по 30 ноября 2015 года) и после (с 1 декабря 2015 года по 30 сентября 2017 года) внедрения вируса Зика, разделяя данные на группы в зависимости от расположения места жительства матери — на высоте выше или ниже 2200 м над уровнем моря. Авторы использовали анализ прерванных временных рядов Пуассона, статистическое моделирование и графический анализ.

**Результаты:**

Оценка предполагаемого количества случаев симптоматической инфекции, которые могли возникнуть у населения Мексики в целом между 25 ноября 2015 года и 20 августа 2016 года, равна 60 172 случаям и оказалась в 7,3 раза выше, чем соответствующее количество зарегистрированных случаев. Ежемесячное количество случаев микроцефалии на 100 000 родившихся живыми младенцев было значительно выше после внедрения вируса, чем до этого (коэффициент заболеваемости, IRR (incidence rate ratio): 2,9; 95%-й доверительный интервал, ДИ: от 2,3 до 3,6), особенно среди младенцев, чьи матери проживают на высоте ниже 2200 м над уровнем моря (IRR: 3,4; 95%-й ДИ: от 2,9 до 3,9).

**Вывод:**

Эпидемия в Мексике, по-видимому, имеет намного больший масштаб, чем указано в оценках, основанных исключительно на подсчетах лабораторно подтвержденных случаев, и связана со значительным повышением риска микроцефалии.

## Introduction

An epidemic of Zika virus infection emerged in the Americas in 2015.^1^ Although, in general, such infection has mild symptoms,^1^ extensive evidence indicates that it can lead to congenital anomalies and neurological disorders.^2^^–^^7^ In 2016, the International Health Regulations (2005) Emergency Committee recommended that, to detect, monitor and respond to an epidemic, affected countries should report data on the occurrence of Zika virus infection and its complications in a timely manner.^8^^,^^9^ Although most countries have since provided weekly counts of suspected and confirmed cases and age-specific attack rates,^10^^–^^12^Mexico has only reported symptomatic cases that have been confirmed, using a polymerase chain reaction diagnostic test, within five days of symptom onset.^13^^–^^15^

In settings where case detection, diagnosis and reporting follow standardized surveillance protocols, the regular recording of the numbers of confirmed cases may help to identify the emergence of an epidemic, sketch the epidemic curve, characterize the spatiotemporal trends in dispersion, recognize the end of transmission and monitor the effect of any control interventions. However, to estimate the magnitude of an epidemic, we need to know not just the number of confirmed cases but also the number of suspected cases, the proportion of the suspected cases that are tested and the mean sensitivity of the test being used.

We estimated the magnitude of the epidemic of Zika virus infection in Mexico, from the number of confirmed cases among pregnant women. We also compared the incidence of congenital microcephaly, as reported, routinely, in the national birth-certificate database, over two periods: before and after Zika virus was confirmed to be circulating in Mexico.

## Methods

### Symptomatic infection

We estimated the total number of symptomatic cases of Zika virus infection that had occurred in Mexico between the day on which the first confirmed Mexican case was reported, i.e. 26 November 2015,^15^ and 20 August 2016. We based these estimates on the reported numbers of confirmed cases among pregnant women that were reported – to the World Health Organization (WHO) by the Mexican Ministry of Health,^16^^,^^17^ over the same period. We assumed that, over our study period: (i) all pregnant women showing the symptoms of Zika virus infection were tested for the virus, as recommended in national guidelines, and (ii) the prevalence of infection among pregnant women was the same as that among the total population. To convert the absolute numbers of cases to the number of confirmed cases per 100 000 pregnancy-months, we estimated the number of pregnancy-months during the study period. For this, we first calculated the relevant annual number of live births by multiplying the numbers of Mexican women aged 15–49 years at the start of the study period, which were projected from the relevant data recorded in the 2010 national census and stratified in five-year age intervals,^18^ by their corresponding age-specific fecundity rates.^19^ Next, to estimate the corresponding annual number of pregnant women, the total number of live births was increased by 11.5% to account for the pregnancies that ended in stillbirth or abortion. Stillbirth and abortion rates were estimated directly from national hospital discharge and stillbirth registries, respectively.^20^^,^^21^ To account for the monthly variation in birth rates, we distributed our estimation of the annual number of pregnancies according to the monthly distribution of births in the national birth-certificate registry.^22^ With this information, we estimated the number of pregnancy-months during the study period. Finally, we allocated the pregnancy-months into two strata of risk of exposure to Zika virus, according to whether the pregnant woman’s place of residence was at least 2200 m above sea level. In Mexico, the *Aedes aegypti* mosquitoes that act as vectors of Zika virus are only endemic at altitudes of less than 2200 m above sea level.^23^ We estimated altitudes of residence from the altitudes recorded, for each human settlement in Mexico, in the 2010 national census.^24^

The total number of symptomatic infections with Zika virus that occurred in Mexico over our study period was estimated by multiplying the estimated cumulative incidence of symptomatic infection among pregnant women by an estimate of the total population living at altitudes below 2200 m. The latter estimate was a projection based on data collected in the 2010 national census. We also estimated the total number of asymptomatic infections, assuming that in Mexico, as observed in an outbreak in the Federated States of Micronesia,^25^ there were 4.5 asymptomatic infections for each symptomatic one.

### Microcephaly

Using cases of microcephaly reported in the national birth-certificate database,^22^ we estimated the annualized incidence of congenital microcephaly for two periods that we categorized, in terms of Zika virus presence in Mexico, as before (1 January 2010–30 November 2015) and after (1 December 2015–30 September 2017). Using the same database, we also created a time-series of the corresponding monthly incidences of microcephaly.

Mexico’s birth-certificate database contains information on all births registered in Mexico since 2010. The recorded variables include the sex and birth weight of the child, the age and place of residence of the mother and the date and place of birth. The attending physician’s descriptions of any physical anomalies identified during physical examination of the baby are also recorded, as is the corresponding code from the 10th revision of the *International* *statistical* *classification* *of* *diseases* *and related* *health* *problems* (ICD-10).^26^ Any anomaly coded Q02.X was assumed to represent a case of congenital microcephaly. We used such cases to estimate incidences of microcephaly, as the numbers of cases per 100 000 live births.

We then used a mathematical model, based on a Poisson interrupted time series,^27^ to test the hypothesis that the incidence of microcephaly in Mexico increased significantly after Zika virus was first detected in the country. For this, we used the equation:

*Y_t_* = *B*_0_ + *B*_1_*T* + *B*_2_*X* + offset[log(*N*)]

where *Y_t_* represents the number of cases of microcephaly in the period *t*, *B*_0_ represents the baseline incidence of microcephaly, *B*_1_ is the change in incidence over time, *B*_2_ is an estimate of the incidence rate ratio (IRR) resulting from a comparison of incidence in the period after the virus was introduced with that in the period before introduction, *T* represents the time elapsed since the start of the study period, in months, and *X* is a dichotomous indicator coded 1 for times between 1 December 2015 and 30 September 2017 and 0 for all other times. The monthly number of births (*N*), incorporated as an offset term, allows the incidence of microcephaly to be estimated as cases per 100 000 live births.

We broke down the resultant time-series into its component parts,– i.e. trend, seasonal variation and residual noise or remainder, using a seasonal-trend decomposition procedure based on a locally-weighted regression.^28^ We fitted the time-series to three different generalized linear mixed models. For each, we used time as the sole fixed component, allowing a random intercept by year, and stratified by the elevation of the mother’s place of residence, as in our estimation of the incidence of symptomatic virus infection. We incorporated time as a linear term, as a linear spline or as a cubic spline.^29^ Linear splines allow for a change in the slope of a fitted straight line at predefined points, known as knots. Cubic splines add more flexibility, allowing the data to fit a curve over a specified time-period. Using analysis of variance, we tested the hypothesis that the time trend in reported microcephaly increased monotonically after November 2015. Using Akaike and Bayesian information criteria, we then selected the best fitting model.

## Results

### Symptomatic infection

We estimated that, between 25 November 2015 and 20 August 2016, there were 2 639 451 pregnancies in Mexico, including 1 439 933 in the 14 states in which pregnant women with confirmed Zika virus infection were reported. Since, over the same period, 953 pregnant women with confirmed symptomatic Zika virus infection were reported in Mexico, the corresponding cumulative incidences of such infection were estimated at 36.11 and 66.18 cases per 100 000 pregnancy-months, respectively (Table 1). When we multiplied the estimated cumulative incidence of symptomatic infection among pregnant women by the total population living at altitudes of less than 2200 m above sea level, we obtained an estimate of 60 172 for the number of symptomatic Zika virus infections that occurred throughout Mexico between 25 November 2015 and 20 August 2016. Our estimate of the corresponding number of asymptomatic infections was 4.5-fold higher, i.e. 270 774.

**Table 1:** Numbers of pregnancies and reported numbers and incidence of confirmed Zika virus infection among pregnant women, Mexico, 25 November 2015–2 September 2016

Area	No. of pregnancies				Confirmed Zika virus infections in pregnant women	
	Total	Among women living at altitudes^a^ of:			No.	Per 100 000 pregnancy-months
		< 2 200 m	≥ 2 200 m			
**State**						
Aguascalientes	41 293	41 249	44		0	0
Baja California	93 541	93 541	0		0	0
Baja California Sur	20 282	20 282	0		0	0
Campeche	25 592	25 592	0		10	39.1
Coahuila	84 214	84 074	140		0	0
Colima	21 390	21 383	6		26	121.6
Chiapas	176 037	170 189	5 848		336	197.4
Chihuahua	107 788	103 672	4 116		0	0
Durango	51 833	48 940	2 893		0	0
Guanajuato	176 667	173 767	2 900		0	0
Guerrero	112 856	111 580	1 276		296	265.3
Hidalgo	86 651	54 358	32 293		1	1.8
Jalisco	232 499	231 016	1 484		2	0.9
México	484 171	18 117	466 054		0	0
Michoacán	140 226	126 445	13 781		8	6.3
Morelos	54 845	53 546	1 300		3	5.6
Nayarit	35 308	35 238	70		0	0
Nuevo León	132 780	132 596	184		1	0.8
Oaxaca	125 074	119 421	5 653		138	115.6
Puebla	198 890	150 656	48 235		0	0
Querétaro	59 333	55 566	3 767		0	0
Quintana Roo	45 936	45 936	0		9	19.6
San Luis Potosí	82 802	82 203	599		0	0
Sinaloa	84 464	84 463	1		0	0
Sonora	83 031	83 031	0		0	0
Tabasco	69 536	69 539	0		25	36.0
Tamaulipas	95 783	95 777	6		0	0
Tlaxcala	39 643	182	39 461		0	0
Veracruz	218 348	211 607	6 741		82	38.8
Yucatán	61 888	61 888	0		16	25.9
Zacatecas	48 328	33 601	14 728		0	0
**Federal district**						
Mexico City	196 254	0	196 254		0	-
**Total**	3 487 284	2 639 451	847 833		953	36.1
**Total in areas reporting cases of Zika virus in pregnant women**	1 503 658	1 435 093	68 565		953	66.4

^a^ above sea level.

Source: Mexican Ministry of Health.^16^^,^^17^

Between 25 November 2015 and 20 August 2016, each of 14 Mexican states reported at least one confirmed case of Zika virus infection. Together, these 14 states represent 38% of the land area and 44% of the national estimated population in 2015. The cumulative incidences of symptomatic confirmed infection among pregnant women ranged from 0.75 case per 100 000 in the northern state of Nuevo León to 265 cases per 100 000 in south-western Guerrero (Table 1). After Guerrero, the states with the highest incidences were the southern states of Chiapas and Oaxaca, with 197.4 and 115.6 cases per 100 000, respectively.

### Microcephaly

**Fig. 1:**
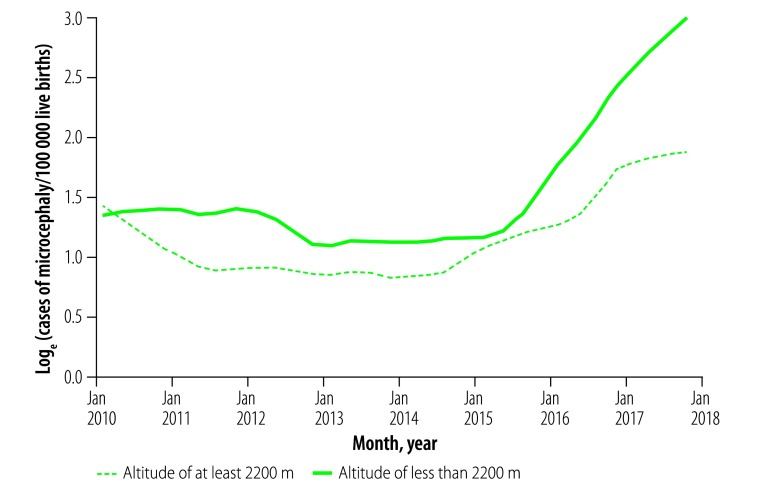
Monthly incidence of congenital microcephaly according to the altitude of the mother’s place of residence, Mexico, January 2010–September 2017

Notes: Based on data in Mexico’s birth-certificate database^22^ and altitudes recorded in the 2010 national census.^24^

We found that, in terms of the number of cases per 100 000 births, the incidence of microcephaly in the period after the Zika virus was introduced in the country was significantly higher than that in the period before introduction. In Mexico, 12.7 million births were registered and 468 cases of congenital microcephaly were reported during the period before Zika virus introduction. The corresponding estimated incidence of microcephaly was 3.7 (95% confidence interval, CI: 3.34 to 4.01) cases per 100 000 births. In contrast, during the period after the Zika virus was introduced, there were 3.7 million births registered and 428 reported cases of microcephaly, giving an estimated cumulative incidence of 11.5 (95% CI: 10.42 to 12.6) cases per 100 000. The Poisson interrupted time-series model indicated a corresponding increase in the rate of microcephaly of about 3-fold (IRR: 2.9; 95% CI: 2.3 to 3.6). As expected, the increase was more pronounced among those living at less than 2200 m above sea level (IRR: 3.4; 95% CI: 2.9 to 3.9) than among those living at higher altitudes (IRR: 2.7; 95% CI: 1.2 to 3.4). Fig. 1 shows the monthly incidences among mothers living below 2200 m and at higher altitudes.

**Fig. 2:**
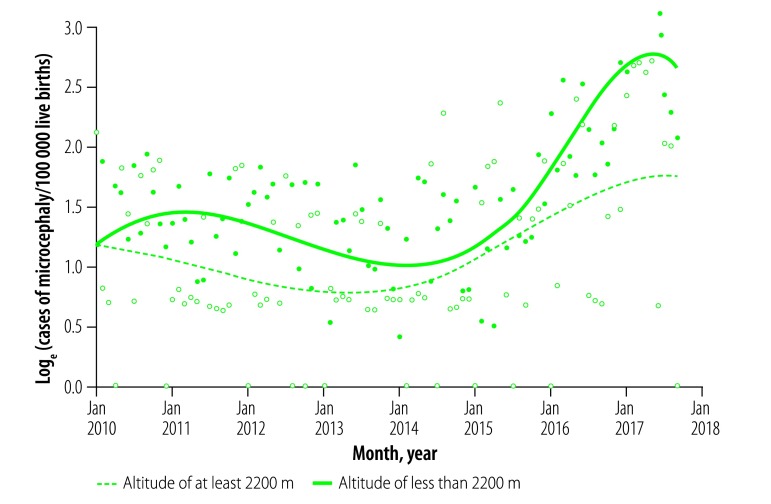
Graphical output of a generalized linear mixed model of a time series for the incidence of congenital microcephaly, Mexico, January 2010–September 2017

Notes: The model incorporated time as a cubic spline to allow the trend slope for incidence of congenital microcephaly to deviate away from a straight line or combination of straight lines. The dots and lines represent the observed and modelled data, respectively. The results for incidence among women living at less than 2200 m above sea level and at higher altitudes are shown separately.

When the goodness-of-fit of each of the three generalized linear mixed models was compared (Table 2), the linear-spline model was found to be a good fit to the data from both altitude categories. The cubic-spline model was, however, only a good fit to the data for populations living at less than 2200 m above sea level (Fig. 2).

**Table 2:** Goodness of fit of three mathematical models to monthly counts of cases of congenital microcephaly, Mexico, 2010–2016

Altitude^a^ of maternal residence	Model**^b^**	Df	AIC	BIC	LogLik	Deviance	*Χ^2^*	
							Value	*P*
≥ 2 200 m	Base model	4	204.8	214.9	−98.38	196.76		
	With linear spline	5	201.9	214.6	−95.96	191.91	4.843^c^	0.027
	With cubic spline	7	201.3	219.0	−93.63	187.27	4.645^d^	0.098
< 2 200 m	Base model	4	162.4	172.5	−77.2	154.39		
	With linear spline	5	156.2	168.9	−73.1	146.19	8.204^c^	0.004
	With cubic spline	7	147.0	164.7	−66.49	132.97	13.22^d^	0.001

AIC: Akaike information criterion; BIC: Bayesian information criterion; Df: degrees of freedom; LogLik: log-likelihood.

^a^ Above sea level.

^b^ Three different mathematical models were used to see if the incidence of congenital microcephaly in Mexico increased significantly after Zika virus was detected in the country. The base model assumed no change in the trend in incidence with time. The models that incorporated time as a linear or cubic spline allowed for changes in the trend slope at fixed points in time and for departures of that slope from one or more straight lines, respectively.

^c^ With one degree of freedom.

^d^ With two degrees of freedom.

## Discussion

We estimated that 60 172 symptomatic infections with Zika virus occurred in Mexico between 25 November 2015 and 2 September 2016. Although we believe that this estimate is conservative, it is still about 40 times higher than indicated by the corresponding incidence rate previously reported, 1.66 cases per 100 000 population,^16^ and almost 30 times higher than the number of confirmed Zika cases reported for Mexico during this time.^17^

Our other estimates indicate a threefold increase in incidence of microcephaly following the confirmed introduction of Zika virus. Much of our mathematical modelling also indicates that the introduction of the virus was positively associated with a significant increase in the monthly incidence of microcephaly, particularly among populations living lower than 2200 m above sea level.

Our national estimate of the number of symptomatic Zika virus infections that occurred per 100 000 pregnancy-months, i.e. 36.11–66.18 cases, appears low compared with the corresponding values reported for Brazil (131.4), Colombia (210.3) and Venezuela (183.4).^16^^,^^17^ However, the Mexican incidence numbers showed considerable geographical variation and the numbers for the states of Guerrero (265.3), Chiapas (197.4), Colima (121.6) and Oaxaca (115.6) were closer to those reported in South America. Given the problem posed by dengue virus,^30^ also transmitted by *Aedes aegypti*,^23^ in the same areas, we were surprised by the relatively low incidences of symptomatic Zika virus infection we recorded among the pregnant women living in the states of Jalisco, Nayarit and Tamaulipas. There may well be underreporting of confirmed cases in these states.

Although we believe that our research provided a reasonable estimate of the magnitude of the Zika virus epidemic in Mexico, it had several limitations. First, we assumed that, during our period of interest, and as recommended in national guidelines, all pregnant women showing the symptoms of Zika virus infection in Mexico were tested for such infection within five days of symptom onset. Given the many resource-poor settings and the many unintended and late-recognized pregnancies^31^ that occur in Mexico, this seems unlikely. Zika virus infection in some pregnant women may have gone undetected because testing did not occur within five days of symptom onset, the women were not aware that they were pregnant and/or the women’s symptoms were so mild that the women did not seek health care. In Mexico, however, pregnant women in their second and third trimesters use health services more intensively than any other population group.^32^ The level of underreporting of symptomatic Zika virus infection during pregnancy may therefore be small. One of Mexico’s largest providers of health care, the *Instituto Mexicano del Seguro Social*, estimates that it tests about 80% of the pregnant Mexican women who present with suspected Zika virus infection and detects the virus in about 27.8% of the women it tests.^33^ If we assume that the prevalence of infection in the untested is the same as that in the tested, and that the test used has a sensitivity of 100%, these figures indicate that 5.5% of the symptomatic infections in pregnant women are never detected. However, as we could not tell if the *Instituto*’s data were nationally representative, we decided not to assume that our estimates of the numbers of symptomatic infections in pregnant women were 5.5% too low.

Another potential source of bias in our approach is our assumption that, compared with other individuals, pregnant women are no more and no less susceptible to Zika virus infection and no more or less likely to develop the characteristic symptoms of such infection once infected. However, the results of a large-scale study of 28 219 non-pregnant symptomatic cases in Puerto Rico indicated that incidence of symptomatic infection among females was markedly higher than that among males: 936 versus 576 cases per 100 000.^34^ These results, and similar data from Brazil,^35^ the Federated States of Micronesia^36^ and Mexico,^15^ indicate that, compared with males, females are more likely to be infected, more likely to develop the characteristic symptoms once infected and/or more likely to be tested once symptomatic, perhaps because of concern about congenital abnormalities. One conclusion of a recent systematic review was that sexual transmission may be responsible for a substantial proportion of cases of Zika virus disease.^37^ In particular, such transmission may act as a maintenance mechanism during periods of low vector density.^38^ Although the topic remains controversial,^39^^,^^40^ we cannot exclude the possibly that pregnancy-related changes in a woman’s immune system enhance her susceptibility to symptomatic Zika virus infection.^41^

Like our estimation of the incidence of symptomatic infection with Zika virus, our evaluation of the temporal trend in the incidence of congenital microcephaly in Mexico also has its limitations. Although a sharp increase in microcephaly incidence, after Zika virus was first confirmed in Mexico, is visible in the birth-certificates data that we examined, this increase, like the similar one reported in Colombia,^42^ cannot be unequivocally attributed to the arrival of the virus. Instead, it may represent a random co-occurrence or an increase in the percentage of cases reported, e.g. as a result of increased awareness of the possible association of microcephaly with Zika virus, or it may have another, as-yet unidentified cause. However, the fact that, in our study, the sharp increase was more apparent in the population living lower than 2200 m above sea level, i.e. where the known vector is endemic,^23^ adds support to the theory that Zika virus was the main or only cause of the increase. In Fig. 1 the shape of the curve showing the monthly incidence of congenital microcephaly among women living below 2200 m above, suggests that Zika virus may have been circulating in Mexico for at least a few weeks before the first case of human infection with the virus was confirmed in the country, in November 2015. This possibility has been mentioned before.^43^^,^^44^

The cases of microcephaly recorded on birth certificates are the result of passive reporting by the physicians attending the births and are not subject to any form of validation. Under these conditions, misclassification of the type of congenital anomaly present, if any, and failure to notice and/or record a mild congenital anomaly that is present may be quite common.^45^^–^^48^ Evaluation of the accuracy and validity of the Mexican birth-certificate database appears not to been done in this regard. Our estimate of the incidence of microcephaly in Mexico before Zika virus was introduced, i.e. 3.7 cases per 100 000 live births, appears to be relatively low when compared with the rates reported for Europe^2^ and the United States of America,^49^ i.e. 19 and 20–120 cases per 100 000 live births, respectively. Plausible explanation is that most of the microcephaly cases reported on birth certificates in Mexico are the more severe cases that are clinically obvious and that many milder cases go unreported.

According to our estimates, there were 177 more Mexican cases of microcephaly after the virus was introduced than might have been predicted from the trend before introduction, i.e. 177 cases that could be, tentatively, attributed to maternal infection with Zika virus. Based on this value and assuming a range of 3.4–10 cases of microcephaly per 1000 pregnant women infected with Zika virus,^2^ we can estimate the number of pregnant women in Mexico who were infected with Zika virus by 30 September 2017 to lie between 18 000 and 52 000. In a previous mathematical simulation, the number of Zika-virus-attributable cases of microcephaly that would have occurred in Mexico by 10 December 2017 was predicted to lie between 314 and 1493.^44^ By epidemiological week 30 of 2017, however, Mexico had only formally reported 15 confirmed cases of such microcephaly.^17^ Compared with the excess of 177 microcephaly cases apparent in the birth-certificate database, this number seems rather small. This number probably illustrates the limitations of using only confirmed cases among pregnant women to monitor the complications of the epidemic of Zika virus infection.

In conclusion, a surveillance system based solely on confirmed cases is unlikely to capture the magnitude of an epidemic of Zika virus infection and needs to be supported by the routine collection and analysis of other data. Despite the limitations of our assessment, our results hint at the true magnitude of the epidemic in Mexico and of the full burden that the epidemic does and may place on Mexico’s health system. If the trends we observed continue, the annual number of infants born with microcephaly in Mexico may continue to rise.

In its response to epidemics of Zika virus and other pathogens, Mexico’s health system needs to strengthen its surveillance and response capacities, improve the quality of its data collection and sharing, develop greater transparency and, whenever possible, adopt more accurate and sensitive diagnostic tests.
